# Insights into Early-Pregnancy Mechanisms: Mast Cells and Chymase CMA1 Shape the Phenotype and Modulate the Functionality of Human Trophoblast Cells, Vascular Smooth-Muscle Cells and Endothelial Cells

**DOI:** 10.3390/cells11071158

**Published:** 2022-03-29

**Authors:** Ningjuan Zhang, Anne Schumacher, Beate Fink, Mario Bauer, Ana Claudia Zenclussen, Nicole Meyer

**Affiliations:** 1 Department of Environmental Immunology, UFZ-Helmholtz Centre for Environmental Research Leipzig-Halle, 04318 Leipzig, Germany; ningjuan.zhang@ufz.de (N.Z.); anne.schumacher@ufz.de (A.S.); beate.fink@ufz.de (B.F.); mario.bauer@ufz.de (M.B.); ana.zenclussen@ufz.de (A.C.Z.); 2 Perinatal Immunology, Saxonian Incubator for Clinical Translation (SIKT), Medical Faculty, University Leipzig, 04103 Leipzig, Germany

**Keywords:** chymase CMA1, mast cells, spiral arteries, extravillous trophoblast cells, endothelial cells, vascular smooth-muscle cells, metalloproteinases, pregnancy

## Abstract

Spiral-artery (SA) remodeling is a fundamental process during pregnancy that involves the action of cells of the initial vessel, such as vascular smooth-muscle cells (VSMCs) and endothelial cells, but also maternal immune cells and fetal extravillous trophoblast cells (EVTs). Mast cells (MCs), and specifically chymase-expressing cells, have been identified as key to a sufficient SA-remodeling process in vivo. However, the mechanisms are still unclear. The purpose of this study is to evaluate the effects of the MC line HMC-1 and recombinant human chymase (rhuCMA1) on human primary uterine vascular smooth-muscle cells (HUtSMCs), a human trophoblast cell line (HTR8/SV-neo), and human umbilical-vein endothelial cells (HUVEC) in vitro. Both HMC-1 and rhuCMA1 stimulated migration, proliferation, and changed protein expression in HUtSMCs. HMC-1 increased proliferation, migration, and changed gene expression of HTR8/SVneo cells, while rhuCMA treatment led to increased migration and decreased expression of tissue inhibitors of matrix metalloproteinases. Additionally, rhuCMA1 enhanced endothelial-cell-tube formation. Collectively, we identified possible mechanisms by which MCs/rhuCMA1 promote SA remodeling. Our findings are relevant to the understanding of this crucial step in pregnancy and thus of the dysregulated pathways that can lead to pregnancy complications such as fetal growth restriction and preeclampsia.

## 1. Introduction

The process of spiral-artery (SA) remodeling is a crucial stage in a successful pregnancy. SAs are maternal arteries that transport maternal blood to the intervillous space of the placenta to nourish the fetus. During early pregnancy, SAs are transformed from low-flow, high-resistance into high-flow, low-resistance vessels to make sure the fetus receives enough nutrients and blood [1,2,3,4,5,6,7]. Insufficient SA remodeling results in pregnancy failure or adverse pregnancy complications, such as abortion, preeclampsia, and intrauterine growth restriction (IUGR) [8,9,10].

SA remodeling is the result of complex and highly orchestrated steps. Initial changes to the remodeling process include vacuolation of endothelial cells, followed by separation and dedifferentiation of vascular smooth-muscle cells (VSMCs) [11,12,13]. Depending on the environment, VSMCs can switch from a contractile to a synthetic phenotype and vice versa. These phenotypic changes lead to altered migration and proliferation behavior and can be monitored by means of contractile markers such as α-smooth-muscle actin, smoothelin, calponin 1, H-caldesmon, and myosin heavy-chain or synthetic markers such as fibronectin and collagen I [14,15,16]. During SA remodeling, VSMCs adopt a synthetic phenotype, leading to increased migration and apoptosis, accompanied by their loss from the vessel wall. These initial processes, endothelial-cell vacuolation and separation/dedifferentiation of VSMCs, are supported by maternal immune cells [17,18]. The next step in the remodeling process is the invasion of fetal extravillous trophoblast cells (EVTs) into the SAs.

The migration of EVT from the fetal villi to SA vessels is regulated by cell–cell and cell–matrix interactions [19,20]. Extensive extracellular matrix breakdown (ECM) is required to achieve the changes in the vessel wall. Proteins of the matrix-metalloproteinase (MMPs) family play important roles in the breakdown of the ECM in normal physiological processes, such as tissue remodeling. MMPs are mainly regulated by tissue inhibitors of metalloproteinases (TIMPs). The balance of MMPs and TIMPs has been implicated in the migration and invasion of EVTs [21,22,23,24,25]. EVT invasion is associated with further disorganization and disappearance of VSMCs from the vessel wall. Finally, EVTs embedded in a fibrinoid-rich matrix replace endothelial cells and VSMCs. The result of a successful remodeling process is the loss of the musculoelastic vessel wall, which enlarges the arterial lumen and reduces the blood-flow resistance. This ensures a constant blood supply at an optimal flow rate into the placenta.

Several immune-cell populations such as uterine natural killer cells (uNKs), regulatory T cells, dendritic cells, and macrophages support the SA-remodeling process at the feto-maternal interface [18,26,27,28,29]. In recent years, it has been shown that mast cells (MCs) also facilitate SA remodeling and fetal growth [30,31,32,33]. MCs, best known for their function in allergic diseases, release a batch of mediators upon activation. The major mediators released in large amounts upon MC activation are chymases. While mouse MCs express multiple chymases such as mast-cell protease (Mcpt) 1, 2, 4, and 5, human MCs express only one chymase, which is called CMA1. The absence of MCs, and more specifically chymase-producing cells, negatively impact SA remodeling, as well as placental and fetal growth, as shown in different mouse models [34,35].

Efficient uterine-artery remodeling is crucial to a successful pregnancy. However, the mechanisms by which MCs or chymases support SA remodeling are unknown. In this study, we aim to investigate the potential effects of MCs and recombinant chymase CMA1 (rhuCMA1) on specific steps of the SA-remodeling process, by studying the phenotype and functionality of VSMCs, EVTs, and endothelial cells.

## 2. Materials and Methods

### 2.1. Primary Cells and Cell Lines

Human uterine primary smooth-muscle cells HUtSMC (PromoCell, Heidelberg, Germany), derived from the human myometrium, were grown in the Medium Kit (Cell Biologics, Chicago, IL, USA) with 10% FBS, 0.1% insulin, 0.1% epidermal growth factor (EGF), 0.1% fibroblast growth factor (FGF) and 1% L-Glutamine. The immortalized human extravillous cytotrophoblast-cell line HTR-8/SVneo (ATCC, Manassas, VA, USA) was cultured in RPMI1640 medium (Gibco, Schwerte, Germany) supplemented with 10% FBS (Pan-Biotech, Aidenbach, Germany), 1% penicillin/streptomycin (P/S, Invitrogen, Düsseldorf, Germany), 1 mmol/L sodium-pyruvate (Sigma, Steinheim, Germany), 0.1 mM/L MEM nonessential acids (Gibco, Schwerte, Germany), and 10 mmol/L HEPES (Gibco, Schwerte, Germany). The human mast-cell line HMC-1 (kindly provided by Dr. J H Butterfield, Rochester, MN, USA) was maintained in IMDM medium (Gibco, Schwerte, Germany) supplemented with 10% FBS and 1% P/S. HUVECs (ATCC, Manassas, VA, USA) were maintained in Medium 199 (Gibco, Schwerte, Germany) supplemented with 20% FBS (or 0.4% for low serum medium), 1% P/S, 12 µg/mL endothelial-cell-growth supplement (ECGS, PromoCell, Heidelberg, Germany), 5 U/mL Heparin, 2 mmol/L L-glutamine, 5 µg/mL ascorbic acid, and 5 µg/mL glutathione.

All cells were cultured in T25 or T75 flasks at 37°C with 5% CO_2_ and humidified atmosphere. The medium was replaced every 2–3 days and cells were detached with 0.05% trypsin-EDTA (Life Technologies, Darmstadt, Germany) when they reached 90% confluence.

### 2.2. Reagents

Recombinant human chymase (rhuCMA1, Sigma, Steinheim, Germany), was used in different concentrations (3 ng/mL, 30 ng/mL, 300 ng/mL). Calcium ionophore A123187 (Sigma, St Louis, MO, USA) was used in a concentration of 1 µM.

### 2.3. Immunofluorescence

2 × 10^4^ HUtSMC cells were seeded in 18 × 18 mm cover slides in 6-well cell-culture plates and either cultured with HMC-1 cells (cell-to-cell ratio 1:1 and 1:5) in 0.4 µm pore-size inserts (Corning, Amsterdam, Netherland) using HMC-1 medium or cultured with HUtSMC growth medium and stimulated with rhuCMA (3 ng/mL, 30 ng/mL, 300 ng/mL) for 24 h. The medium was removed and the cells were rinsed with PBS and fixed with 4% paraformaldehyde for 10 min. The cells were permeabilized in 0.5% Triton X-100 for 3 min and blocked in 1% BSA for 30 min. The slides were incubated with the primary antibody to fibronectin (polyclonal, 1:100, ab2413, Germany) and collagen I (monoclonal, 1:500, ab260043, Germany) for 1 h at RT; for calponin 1 staining, the slides were incubated with primary antibody to calponin 1 (monoclonal, 1:500, ab46794, Germany) at 4 °C overnight. After washing, the slides were incubated with goat-anti-rabbit IgG (H + L) secondary antibody Alexa Fluor 488 (polyclonal, 1:1000, ab150077, Germany) for 1 h, and then mounting medium with DAPI (Vector #H-1200) was used. Images were captured using a fluorescence microscope (Zeiss, Jena, Germany) with a 20× objective and staining intensity was analyzed with Image J 1.53f software (NIH, Rockville, MD, USA). Integrated density (IntDen, total value of all pixels within a region) was measured.

### 2.4. Proliferation and Viability Assay

4 × 10^4^ HTR-8/SVneo or 6 × 10^4^ HUtSMC cells per well were seeded in 2 mL cell HTR-8/SVneo or HUtSMC cell-growth medium in 6-well plates. After the cells had become confluent overnight, the medium was replaced by growth medium supplemented with rhuCMA1 in different concentrations (3 ng/mL, 30 ng/mL, 300 ng/mL). In another experimental setting, HTR-8/SVneo or HUtSMCs cells were cocultured with HMC-1 cells (cell-to-cell ratio 1:1 and 1:5) using 0.4 µm pore-size inserts (Corning, Amsterdam, The Netherland) and HMC-1 cell growth medium. After 24 h, 48 h, and 72 h HTR-8/SVneo or HUtSMCs cells were harvested and stained with 0.4% Trypan blue (Sigma, Steinheim, Germany) and living/dead cells were counted. Afterwards, HTR-8/SVneo-cell pellets were collected and stored at −80 °C for RNA isolation. Cell proliferation and viability rates were calculated.

### 2.5. Scratch Assay

6 × 10^4^ HTR-8/SVneo cells per well were plated in 12-well plates. After 16 h, the scratches were generated with a 100 µL pipette tip. HTR-8/SVneo medium supplemented with rhuCMA1 in different concentrations (3 ng/mL, 30 ng/mL, 300 ng/mL) was then added. In another experimental setup, scratched HTR-8/SVneo cells were cocultured with HMC-1 (cell-to-cell ratio 1:1 and 1:5) using 0.4 µm pore-size inserts and HMC-1 cell-growth medium. HTR-8/SVneo cells alone served as control. Images of scratch areas were taken after 6 h, 12 h, and 24 h with the light microscope and Axio Vision software version 4 (Zeiss, Jena, Germany).

### 2.6. Transwell-Migration Assay

The transwell-migration assay was conducted using 8 µm pore-size inserts (Corning, New York, NY, USA) in 24-well plates. HTR-8/SVneo cells or HUtSMC cells were starved in serum-free medium for 16 h. 1 × 10^5^ HTR-8/SVneo cells or 5 × 10^4^ HUtSMC cells were then seeded in 100 µL serum-free medium in the upper part of the transwell chamber. In the lower part of the chamber, either HMC-1 cells (cell-to-cell ratio 1:1 and 1:5) in complete HMC-1 growth medium or HTR-8/SVneo complete-growth medium supplemented with rhuCMA (3 ng/mL, 30 ng/mL, 300 ng/mL) was added. A medium with 10% FBS served as the control. After an incubation time of 24 h, cells on the transwell membrane were fixed appropriately with 70% ethanol and stained with 0.2% crystal violet (Sigma, Steinheim, Germany). The nonmigrated cells on the upper side of the membrane surface were removed with cotton swabs. Migrated cells on the underside of membrane were photographed with an inverted microscope and Axio Vision software version 4 (Zeiss, Jena, Germany, magnification 20×) and five fields in each membrane were counted. Each experiment was carried out in duplicate.

### 2.7. Flow Cytometry

HTR-8/SVneo cells were cultured in 96-well plate in 100 µL HTR-8/SVneo cell-culture medium with or without rhuCMA1 (3 ng/mL, 30 ng/mL, 300 ng/mL). In another experimental setup HTR-8/SVneo cells were cocultured directly in 300 µL HMC-1 growth medium with HMC-1 cells (cell-to-cell ratio 1:1 and 1:5) in 48-well plates and harvested after 24 h. After fixation and permeabilization for intracellular staining, HTR-8/SVneo cells were stained for 30 min at 4 °C with the anti-human antibodies listed in Table 1. Flow-cytometry measurements were performed with the Attune NxT flow cytometer (Thermo Fisher, Bremen, Germany) and analyzed with Attune NxT software.

### 2.8. RNA Isolation, cDNA Synthesis and Quantitative Real-Time PCR

RNA isolation: HTR8/SVneo-cell pellets were collected from the proliferation and viability assay as described above and stored at −80 °C until RNA extraction. Total RNA was extracted using RNAeasy Plus Mini Kit (Qiagen, Hilden, Germany) according to the manufacturer’s instructions. RNA purity was quantified by measuring the UV absorbance at 260 nm, and a quality check was performed by measuring the absorbance at 280 nm. RNA samples were then stored at −80 °C.

cDNA synthesis: Isolated RNA (2 µg) was incubated with oligo dTs and RNAse-free water for 10 min at 75 °C and after 2 min on ice; the marked mRNA was incubated with dNTPs (2.5 mmol/mL), DNAse (2 U/µL), and RNAse (40 U/µL) in a reaction buffer for 30 min at 37 °C. DNAse was inactivated for 5 min at 75 °C and the samples were incubated on ice for 2 min. Reverse transcriptase and RNAse inhibitor were added and cDNA was synthesized at 42 °C for 60 min. Reverse transcriptase was inactivated at 94°C for 5 min and the cDNA samples were stored at −20 °C.

Real-time polymerase chain reaction (RT-PCR): Intron-spanning primers were designed and universal probe-library probes (UPL) were selected by the Universal Probe Library Assay Design Center (http://qpcr.probefinder.com/organism.jsp (accessed on 20 July 2021), currently not available). The cycling program consisted of 95 °C for 5 min, followed by 50 cycles of 95 °C for 15 s, 60 °C for 1 min, and 72 °C for 30 s on a Light Cycler 480 (Roche Applied Science, Mannheim, Germany). PCR was performed with FastStart Universal Probe Master Mix (Roche). All reactions were performed in duplicate. β-glucuronidase (GUSB) was used as a reference gene and relative gene expression was calculated by the 2^−ΔCT^ method. The qPCR primers used are listed in Table 2.

### 2.9. LEGENDplex Multiplex Assay and ELISA

To determine the protein secretion of HTR-8/SVneo cells after coculture with HMC-1 cells (cell-to-cell ratio 1:1 and 1:5) or treatment with rhuCMA1 (3 ng/mL, 30 ng/mL, 300 ng/mL), HTR-8/SVneo supernatant from cells used in the flow-cytometry experiments was collected. LEGENDplex multiplex assay (Biolegend, London, UK) was applied to measure MMP2, MMP9, TIMP1, and TIMP2 according to manual instructions (see Table 3). TIMP3 was measured via Human TIMP3 ELISA Kit (Abcam, Cambridge, UK) according to manual instructions.

To confirm CMA1 secretion of HMC-1 cells, the supernatant of 1 × 10^6^ HMC-1 cells stimulated with or without Calcium Ionophore A23187 (Sigma, C7522, 1 µM) for 24 h was analyzed with the Human chymase Elisa Kit (Biotrend, Köln, Germany) according to manual instructions.

### 2.10. Endothelial-Tube-Formation Assay

Two days prior to the assay, HUVECs were thawed or transferred into a new cell-culture flask. One day before the assay, normal-growth medium was replaced by low-serum medium. 24 h later, 50 µL growth-factor-reduced matrigel (10.3 mg/mL, Corning™, New York, NY, USA) per well was loaded into a 96-well plate and incubated for 30 min in the CO_2_ incubator. 1.5 × 10^4^ HUVECs were mixed with 100 µL low-serum medium with or without rhuCMA1 (3 ng/mL, 30 ng/mL, 300 ng/mL) and placed in each well. Complete medium served as the positive control, while low-serum medium served as the negative control. After an incubation time of 4 h, images were taken with the light microscope (Zeiss, Germany, magnification 20×). The tube network was then quantified for the number of nodes, segments, master segments, meshes, and master junctions with Image-J Angiogenesis Analyzer 1.53f.

### 2.11. Statistical Analysis

Statistical data analysis was performed using GraphPad Prism software version 8.0 (GraphPad, Statcon, Witzenhausen, Germany). Data from the immunofluorescence assay, proliferation and viability assays, scratch assays, transwell-migration assays, and tube-formation assay are presented as a mean with SEM. Data from the flow cytometry and RT-PCR assays are presented as a median. Data was tested with the Shapiro–Wilk test for normal distribution. Group comparisons were performed using the one-way analysis of variance (ANOVA) test when data were normally distributed, otherwise the Friedman test was used. Statistical significance was defined as *p* < 0.05. All results were confirmed in at least three independent experiments.

## 3. Results

### 3.1. Phenotypic Switch of HUtSMCs in Response to HMC-1 or rhuCMA1

**Figure 1:**
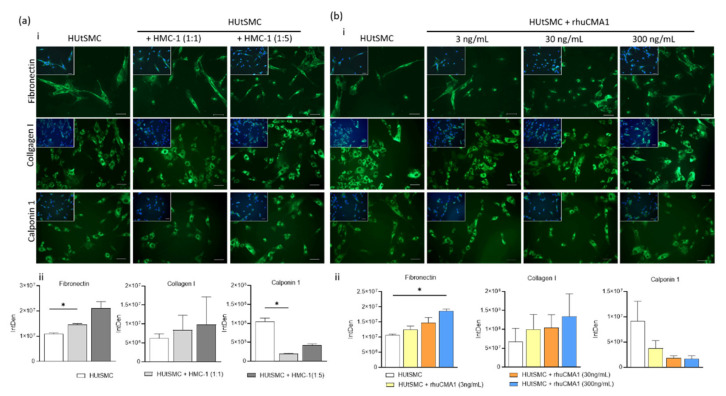
HMC-1 reduces calponin 1 and induces fibronectin expression, while rhuCMA1 induces fibronectin expression in HUtSMCs. Fibronectin, calponin 1 and collagen I expression in HUtSMCs after coculture with HMC-1 (**a**) at different cell-to-cell ratios (HUtSMC:HMC-1 ratio 1:1 or 1:5) or treatment with rhuCMA1 (**b**) at different concentrations (3 ng/mL, 30 ng/mL, 300 ng/mL) at 24 h was detected by immunofluorescence staining and analyzed with ImageJ software (**a**(**ii**),**b**(**ii**)). Representative images are shown in ((**a**(**i**),**b**(**i**)) scale bar 200 µm). The results are presented as a mean with SEM. Statistical analysis was performed using the one-way ANOVA test (* *p* < 0.05). HUtSMC: Human uterine vascular smooth-muscle cells; rhuCMA1: recombinant human chymase.

VSMCs are not terminally differentiated. They are able to switch their phenotype between the quiescent contractile and the migratory synthetic phenotype, a process that can be tracked through the expression of distinct marker proteins [14]. To examine the effect of HMC-1 and rhuCMA1 in terms of the HUtSMCs phenotype switch, the expression of the synthetic markers fibronectin and collagen I and the contractile marker calponin1 was analyzed in HUtSMCs after coculture with HMC-1 cells or stimulation with rhuCMA1. The results show that the expression of the synthetic marker fibronectin, measured as integrated density (IntDen), significantly increased after 24 h exposure to HMC-1 cells (cell-to-cell ratio 1:1, *p* < 0.05, Figure 1a(ii)) or the highest concentration of rhuCMA1 (300 ng/mL, *p* < 0.05, Figure 1b(ii)). While the expression of the collagen I was not changed by HMC-1 (Figure 1a(ii)) or rhuCMA1 (Figure 1b(ii)), coculture of HUtSMCs and HMC-1 cells (cell-to-cell ratio 1:1) led to significantly decreased expression of the contractile marker calponin 1 (*p* < 0.05, Figure 1a(ii)). Representative images are shown in Figure 1a(i),b(i).

To confirm that HMC-1 cells were capable of secreting CMA1, we quantified the supernatant of unstimulated and stimulated HMC-1 cells for CMA1 via ELISA. While unstimulated HMC-1 cells did not secrete CMA1 into the supernatant (or concentration was outside of the detectable range), stimulation of HMC-1 cells with calcium ionophore A23187 resulted in the secretion of CMA1 into the supernatant (Supplementary Figure S1).

### 3.2. HMC-1 and rhuCMA1 Induce Proliferation of HUtSMCs

**Figure 2:**
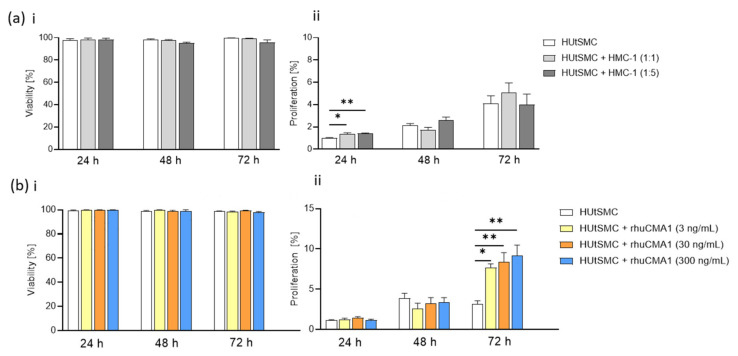
HMC-1 and rhuCMA1 increase HUtSMC proliferation. Viability (**a**(**i**)) and proliferation (**a**(**ii**)) of HUtSMC cocultured with HMC-1 cells (HUtSMC: HMC-1 ratio 1:1 and 1:5) at 24/48/72 h. Viability (**b**(**i**)) and proliferation (**b**(**ii**)) of HUtSMC cells treated with rhuCMA1 (3 ng/mL, 30 ng/mL, 300 ng/mL) at 24/48/72 h. The results are presented as a mean with SEM. Statistical analysis was performed with the one-way ANOVA or Friedman test (* *p* < 0.05, ** *p* < 0.01). HUtSMC: Human uterine vascular smooth-muscle cells; rhuCMA1: recombinant human chymase.

During immunofluorescence experiments, analyzing contractile and synthetic proteins, we discovered an increased quantity of HUtSMCs after coculture with HMC-1 or rhuCMA1. Therefore, proliferation was analyzed in subsequent experiments. To investigate whether HMC-1 or rhuCMA1 affect on HUtSMC proliferation or viability, we conducted a viability and proliferation assay by coculturing HUtSMC cells with HMC-1 or stimulation with rhuCMA. The results show that neither HMC-1 nor rhuCMA1 altered the viability of HUtSMCs (Figure 2a(i),b(i)), but that their proliferation increased significantly after coculture with HMC-1 at 24 h (cell-to-cell ratio 1:1, *p* < 0.05; cell-to-cell ratio 1:5, *p* < 0.01, Figure 2a(ii)). All three rhuCMA1 concentrations increased the HUtSMCs’ proliferation rate significantly at 72 h (3 ng/mL, *p* < 0.05; 30 and 300 ng/mL, *p* < 0.01, Figure 2b(ii)).

### 3.3. HMC-1 and rhuCMA1 Attract HUtSMCs in Transwell-Migration Assays

**Figure 3:**
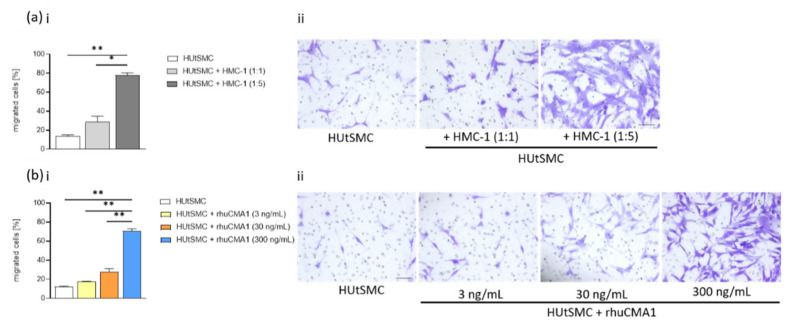
HMC-1 and rhuCMA1 elevate HUtSMC migration. The number of migrated HUtSMC cells cocultured with HMC-1 cells (**a**(**i**)) at different cell-to-cell ratios (HUtSMC:HMC-1 ratio 1:1 and 1:5) or stimulated with rhuCMA1 (**b**(**i**)) at different concentrations (3 ng/mL, 30 ng/mL, 300 ng/mL) at 24 h. Representative images are shown in ((**a**(**ii**),**b**(**ii**)), scale bar 200 µm). Results are presented as mean with SEM. Statistical analysis was performed using one-way ANOVA (* *p* < 0.05, ** *p* < 0.01). HUtSMC: Human uterine vascular smooth-muscle cells; rhuCMA1: recombinant human chymase.

The switch of VSMCs from contractile to synthetic phenotype, is accompanied by an increased VSMC-migration capacity [16]. To examine the effect of HMC-1 and rhuCMA1 on VSMC migration, we performed transwell-migration assays by coculturing HUtSMC cells with HMC-1 cells or stimulating them with rhuCMA1. The number of migrated HUtSMCs significantly increased after 24 h exposure to HMC-1 cells (cell-to-cell ratio 1:5, *p* < 0.01, Figure 3a(i)). Representative images are shown in Figure 3a(ii). To assess whether the increased migration of HUtSMC cells in response to HMC-1 cells was due to the secretion of chymase, the assay was performed with rhuCMA1 instead of HMC-1 cells. The results showed that the highest concentration of rhuCMA1 (300 ng/mL) led to a significant increase (*p* < 0.01) in the percentage of migrated cells after 24 h (Figure 3b(i)). Representative images are shown in Figure 3b(ii).

### 3.4. HMC-1 Supernatant, but Not rhuCMA1, Increases the Proliferation of HTR-8/SVneo Cells

**Figure 4:**
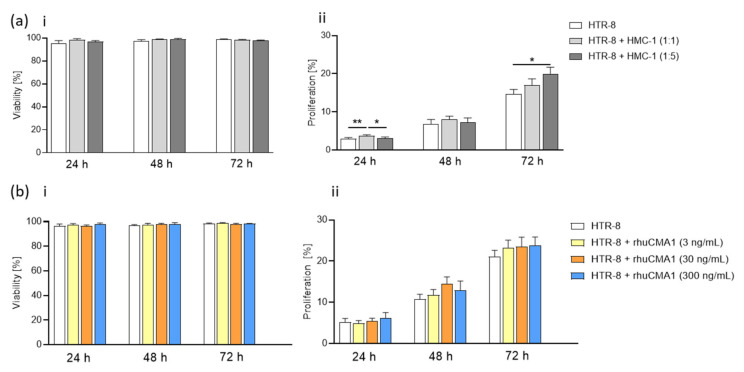
HMC-1 elevates HTR-8/SVneo proliferation. Viability (**a**(**i**)) and proliferation (**a**(**ii**)) of HTR-8/SVneo cocultured with HMC-1 cells (HTR-8/SVneo: HMC-1 ratio 1:1 and 1:5) at 24/48/72 h. Viability (**b**(**i**)) and proliferation (**b**(**ii**)) of HTR-8/SVneo cells treated with rhuCMA1 (3 ng/mL, 30 ng/mL, 300 ng/mL) at 24/48/72 h. The results are presented as a mean with SEM. Statistical analysis was performed with the one-way ANOVA or Friedman test (* *p* < 0.05, ** *p* < 0.01). HTR-8: HTR-8/SVneo; rhuCMA1: recombinant human chymase.

Trophoblast viability and proliferation capacity are important features in the SA-remodeling process [36]. To determine whether MCs themselves or rhuCMA1 influence the viability or proliferation of EVTs, HTR-8/SVneo cells were cocultured with HMC-1 (with inserts) or treated with different concentrations of rhuCMA1. Neither coculturing with HMC-1 cells (Figure 4a(i)) nor stimulation with rhuCMA1 (Figure 4b(i)) influenced the viability of HTR-8/SVneo cells. The proliferation of HTR-8/SVneo cells was significant after 24 h (cell-to-cell ratio 1:1, *p* < 0.01) and 72 h (cell-to-cell ratio 1:5, *p* < 0.05) after co-culture with HMC-1 cells (Figure 4a(ii)). In contrast, there were no significant proliferation differences when HTR-8/SVneo cells were treated with rhuCMA1 (Figure 4b(ii)).

### 3.5. HMC-1 and rhuCMA1 Induce HTR-8/SVneo Migration

**Figure 5:**
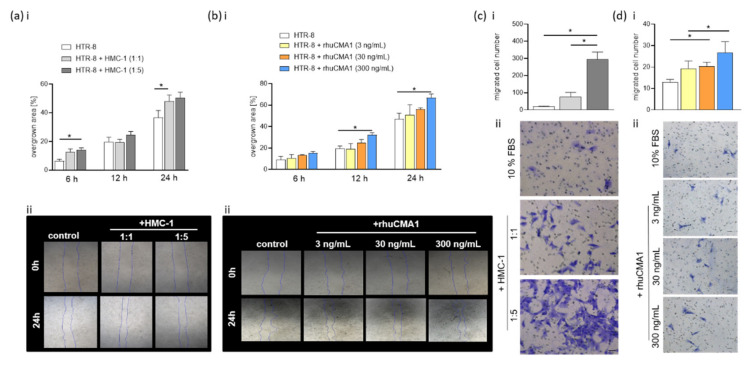
Both HMC-1 and rhuCMA1 increase HTR-8/SVneo migration. The overgrown scratch area (**a**(**i**)) or number of migrated (**c**(**i**)) HTR-8/SVneo cells cocultured with HMC-1 cells (HTR-8: HMC-1 ratio 1:1 and 1:5) or rhuCMA1 (**b**(**i**),**d**(**i**)) at 6/12/24 h. The results are presented as a mean with SEM. Statistical analysis was performed using one-way ANOVA (* *p* < 0.05). Representative images are shown in ((**a**(**ii**),**b**(**ii**),**c**(**ii**),**d**(**ii**)), scale bar 200 µm). HTR-8: HTR-8/SVneo; rhuCMA1: recombinant human chymase.

Trophoblast migration along uterine SAs is an important event in the remodeling process [36]. To assess whether HMC-1 or rhuCMA1 can induce the migration of EVTs, we performed scratch and transwell-migration assays with HTR-8/SVneo cells that were cocultured with HMC-1 cells or rhuCMA1. The scratch assay revealed that when cocultured with inserts, HMC-1 cells accelerate trophoblast migration after 6 h (cell-to-cell ratio 1:5, *p* < 0.05) and after 24 h (cell-to-cell ratio 1:1, *p* < 0.05) (Figure 5a(i)). Representative images are shown in Figure 5a(ii). In contrast to the coculture with inserts, direct coculture of HMC-1 and HTR-8/SVneo cells does not lead to a statistically significant increase in HTR-8/SVneo migration (data not shown).

To establish whether the increased migration of HTR-8/SVneo cells in response to HMC-1 cells is due to the secretion of chymase CMA1, the scratch assay was performed with rhuCMA1. The results show that the highest concentration of rhuCMA1 (300 ng/mL) led to a significant increase (*p* < 0.05) in migration after 12 h and 24 h (Figure 5b(i)). Representative images are shown (Figure 5b(ii)).

To confirm the results of the scratch assay, we performed transwell migration assays. The number of migrated HTR8/SVneo cells significantly increased after 24 h exposure to HMC-1 cells (cell-to-cell ratio 1:5, *p* < 0.05, Figure 5c(i)) and also after 24 h exposure to rhuCMA1 (30 and 300 ng/mL, *p* < 0.05, Figure 5d(i)). Representative pictures are shown in Figure 5c(ii),d(ii).

### 3.6. HMC-1 and rhuCMA1 Downregulate TIMP Expression in HTR-8/SVneo Cells

**Figure 6:**
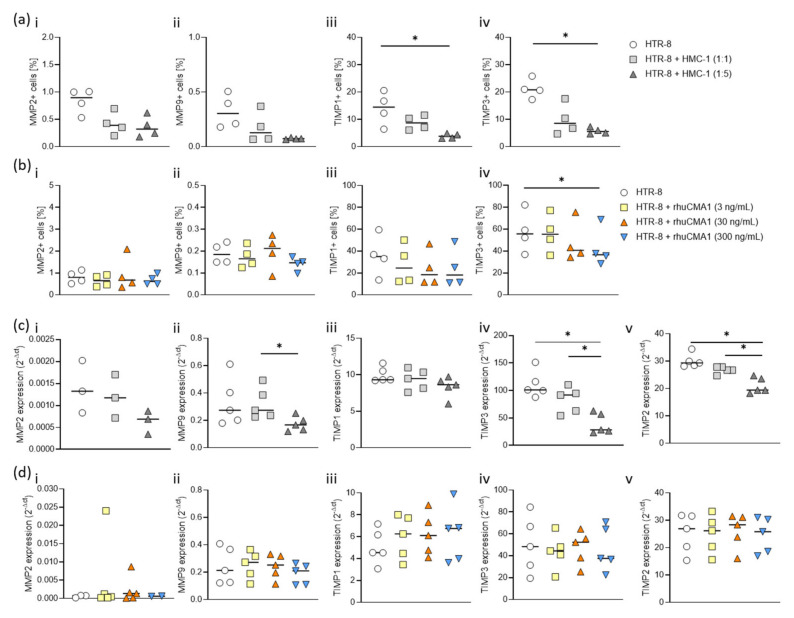
Both HMC-1 and rhuCMA1 downregulate the number of TIMP-positive HTR-8/SVneo cells and TIMP-expression levels. Intracellular protein expression of MMP2 (**i**), MMP9 (**ii**), TIMP1 (**iii**), TIMP3 (**iv**) in HTR-8/SVneo cells after coculture with HMC-1 (**a**) at different cell-to-cell ratios (HTR-8:HMC-1 ratio 1:1 or 1:5) or treatment with rhuCMA1 (**b**) at different concentrations (3 ng/mL, 30 ng/mL, 300 ng/mL) at 24 h was detected by flow cytometry. The mRNA expression of MMP2 (**i**), MMP9 (**ii**), TIMP1 (**iii**), TIMP2 (**iv**) and TIMP3 (**v**) in HTR-8/SVneo cells after coculture with HMC-1 (**c**) at different cell-to-cell ratios (HTR-8:HMC-1 ratio 1:1 or 1:5) or treatment with rhuCMA1 (**d**) at different concentrations (3 ng/mL, 30 ng/mL, 300 ng/mL) at 72 h was measured by RT-PCR. The results are presented as a median. Statistical analysis was performed with the Friedman test (* *p* < 0.05). HTR-8: HTR-8/SVneo; rhuCMA1: recombinant human chymase; MMP: Metalloproteinases; TIMP: Tissue inhibitor of metalloproteinases.

As MMPs and their inhibitors TIMPs are important regulators of EVT migration [25,37,38,39], we analyzed their expression in HTR8/SVneo cells after coculture with HMC-1 or rhuCMA1 via flow cytometry (gating strategy, Supplementary Figures S2 and S3). The coculture of HTR-8/SVneo cells with HMC-1 cells (cell-to-cell ratio 1:5) for 24 h resulted in a significant decrease (*p* < 0.05) in TIMP1- (Figure 6a(iii)) and TIMP3-positive HTR-8/SVneo cells (Figure 6a(iv)). No changes were seen for MMP2 (Figure 6a(i)) and MMP9 (Figure 6a(ii)). Treatment with the highest concentration of rhuCMA1 (300 ng/mL) led to a significant reduction in TIMP3-positive cells (*p* < 0.05, Figure 6b(iv)). No changes were registered for MMP2, MMP9 or TIMP1 (Figure 6b(i–iii)). To determine the gene expression levels of MMPs and TIMPs in HTR-8/SVneo cells in response to HMC-1 or rhuCMA1, we also carried out RT-PCR. While no changes were observed for MMP2 (Figure 6c(i)), MMP9 (Figure 6c(ii)), and TIMP1 (Figure 6c(iii)), mRNA levels of TIMP2 (Figure 6c(iv)) and TIMP3 (Figure 6c(v)) were significantly reduced in HMC-1 cocultured HTR-8/SVneo cells (*p* < 0.05). In contrast, there were no changes observed for MMP2, MMP9, TIMP1, TIMP2, and TIMP3 (Figure 6d(i–v)) when cells were stimulated with chymase. To complement the flow-cytometry data, MMP/TIMP expression was analyzed in the supernatant of HTR-8/SVneo cells cocultured with HMC-1 cells or treated with rhuCMA1. While MMP2 and MMP9 were not detectable in all experimental settings, TIMP3 was significantly decreased in the supernatant of HTR-8/SVneo cells cocultured with HMC-1 (1:5) or treated with rhuCMA1 (300 ng/mL) at 24 h. In contrast, TIMP1 expression was significantly increased in the supernatant of the HTR-8 cells cocultured with HMC-1 (1:5) compared to the control group at 24 h (*p* < 0.01). At 48/72 h, there were no significant differences between both groups. TIMP1 expression was comparable between the rhuCMA1-treated HTR-8/SVneo and the control group at 24/48/72 h (Supplementary Table S1).

### 3.7. rhuCMA1 Induces HUVEC Endothelial-Tube Formation

**Figure 7:**
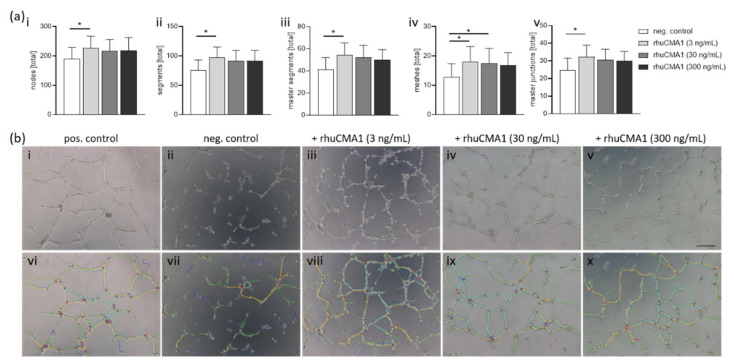
rhuCMA1 induces HUVEC endothelial-tube formation. Tube formation of HUVECs incubated with rhuCMA1 (3 ng/mL, 30 ng/mL, 300 ng/mL) was evaluated after 4 h for number of nodes (**a**(**i**)), segments (**a**(**ii**)), master segments (**a**(**iii**)), meshes (**a**(**iv**)), and master junctions (**a**(**v**)). Representative cell-culture images (**b**(**i**–**v**)) and images analyzed with the Angiogenesis Analyzer tool of Image J (**b**(**vi**–**x**)) are shown (scale bar 200 µm). Results are presented as mean with SEM. Statistical analysis was performed using the Friedman test (* *p* < 0.05). HUVEC: Human umbilical-vein endothelial cells; rhuCMA1: recombinant human chymase.

The generation of a new blood-vessel network in a low-oxygen microenvironment is relevant to SA remodeling [40,41,42]. To evaluate the effect of rhuCMA1 on angiogenesis, we performed an endothelial-tube-formation assay. For this, primary human endothelial HUVEC cells were stimulated with rhuCMA1 and tube formation was analyzed. rhuCMA1 in a concentration of 3 ng/mL significantly increased the number of nodes, segments, master segments, meshes, and master junctions, whereas a concentration of 30 ng/mL rhuCMA1 led to an increased number of meshes (*p* < 0.05, Figure 7a). Representative microscope images (Figure 7b(i–v)) and analyzed images (Figure 7b(vi–x)) are shown.

## 4. Discussion

During early pregnancy, extensive physiological adaptions of the uterine vascular system are needed to ensure an adequate blood supply to the placenta and meet the needs of the growing fetus [43]. Histological and clinical evidence suggests that maternal decidual immune cells probably initiate the SA-remodeling process, which involves important changes in VSMC, EVT, and endothelial phenotype and behavior [17,18,35]. MCs are well known for their role in allergy and inflammation, but also in angiogenesis and matrix-metalloprotease-related remodeling of the cardiovascular system [44,45,46,47]. There are only a few recent studies on the contributions of MCs to pregnancy. Our previous in vivo mouse studies showed that MC deficiency or absence of α-chymase Mcpt5-positive cells results in insufficiently remodeled SAs and subsequent fetal intrauterine growth restriction [34,35]. Moreover, samples from first-trimester pregnant patients showed that uterine MCs are in close contact with extravillous trophoblasts. This close proximity already suggests a function for MCs in early pregnancy. In addition, MCs and rhuCMA1 may stimulate trophoblast migration in human placenta-explant cultures [34]. In this study, we aimed to uncover the mechanisms underlying the positive effects of MCs and rhuCMA1 in processes related to the SA-remodeling process, namely phenotype switch/proliferation/migration of VSMCs, proliferation/migration/altered gene expression of EVTs, and angiogenesis. This work shows that HMC-1 cells and rhuCMA1 modulate the phenotype and the behavior of VSMCs, EVTs, and endothelial cells, shedding light into important milestones during early pregnancy.

We confirmed that HMC-1 cells are capable of secreting CMA1 by using the degranulation agent calcium ionophore A23187. We included the substance in the control experiment as we believe that MCs alone have no reason to degranulate (and secrete CMA1 in the supernatant) without any trigger. In the main experiments, we did not include the degranulation agent as this would not reflect the in vivo situation adequately. We suggest that VSMCs or EVTs themselves are able to activate MCs and provoke degranulation. To analyze whether the observed effects in response to HMC-1 cells are due to the secretion of chymase CMA1, all assays were additionally performed with rhuCMA1.

VSMCs, the main component of arterial medial layers, are not terminally differentiated [16]. In our study, we demonstrate that coculture of HUtSMCs with HMC-1 or rhuCMA1 cause VSMCs to switch to a synthetic phenotype, increasing the expression of the synthetic ECM protein fibronectin and reducing the expression of the contractile protein calponin 1, while there are no significant changes for collagen I. Fibronectin, a 230–270 kDa high-molecular-weight glycoprotein, is present in the ECM of almost all tissues and organs and has also been detected in plasma in soluble form. It is a major regulator of a variety of cellular functions including motility, growth, and differentiation [48]. Collagens are the most abundant proteins in mammals, while type 1 collagen is the most abundant collagen of the human body. Collagens contribute to mechanical properties, organization, and shape of tissues. They interact with cells and regulate their proliferation, migration, and differentiation [49]. Calponin 1 is a 34 kDa protein predominantly found in smooth-muscle tissue. The high-affinity actin-binding protein has been extensively studied for its role in the regulation of smooth-muscle contractility [50].

Changes in ECM composition are often responsible for VSMC phenotype changes. While an ECM composition of laminin, collagen IV, and perlecan promotes a VSMC contractile phenotype, an increased occurrence of osteopontin or fibronectin promotes a VSMC synthetic phenotype [51,52,53,54,55,56]. Interestingly, Lazaar and colleagues have demonstrated that local release of MC chymase affects human-airway smooth-muscle-cell function and airway remodeling by degradation of the VSMC matrix, which is associated with increased release of fibronectin [57]. IL-1b, TNF-α, and prostaglandin D2, expressed by MCs, have also been shown to stimulate phenotypic changes in VSMCs, including the upregulation of fibronectin expression [58,59,60,61].

Our results showed an increased proliferation of VSMCs. Increased HUtSMC proliferation might be due to the dedifferentiated synthetic phenotype with an increased fibronectin expression, as studies have indicated that a switch to a synthetic phenotype is characterized by increased proliferation [51,62]. In our present study, we observed that coculture of HUtSMCs with HMC-1 or rhuCMA increased VSMCs’ migration capacity. This is in line with the fact that synthetic VSMCs increase their migration capacity. Kanta and colleagues showed that chymase promotes VSMC migration in tissue remodeling after injury in dogs [63]. However, we cannot exclude that the higher numbers of migrated cells are due to the fact that the proliferation of VSMCs was also increased by HMC-1 and chymase.

By comparing human placentas from preeclampsia-complicated pregnancies and physiological pregnancies, it has been shown in different studies that the distribution of MCs and concentration of their mediators has an influence on the placental vascular network [64,65,66]. Interestingly, some studies have documented an increased number of MC [64,65], while others [66] documented decreased MC numbers in preeclamptic placentas compared with normal placentas. Since the small number of available studies have produced different results, studies with homogenized study designs are needed in order to determine the role of MCs and their mediators in fundamental reproductive processes. EVTs play an important role in the SA-remodeling process. They actively invade the maternal uterus and modify SAs by replacing VSMCs and endothelial cells within the vessel wall so as to promote placentation. EVT functions are influenced by several factors, including maternal immune cells [67,68]. Our previous study demonstrated that uterine MCs are in close proximity to EVTs in human decidual tissue [34]. In this study, human placental EVT-derived HTR-8/SVneo cells were used to investigate the effects of MCs and chymase CMA1 on EVTs, because placental EVTs have highly conserved characteristics and gene-expression profiles [69,70]. We found a significantly increased proliferation of EVTs after coculture with HMC-1 (using inserts), but not with rhuCMA1. This means that soluble mediators other than CMA1 may be responsible for the observed effect. Additionally, we observed that EVT migration remained unchanged when cells were cocultured directly with HMC-1, but increased significantly when they were cocultured with HMC-1 using inserts or stimulated with rhuCMA1. We confirmed this in two different experimental migration approaches. This indicates that HMC-1 increases EVT migration by means of chymase CMA1 and possibly other soluble factors. Interestingly, direct cell-to-cell contact prevents an increased migration of EVTs. To the best of our knowledge, no studies studied the effect of chymases on EVT migration. Nevertheless, there are numerous studies showing that chymase is a potent chemoattractant for cells other than EVTs, namely eosinophils [71], monocytes and neutrophils [72], and even MCs, presenting a self-amplification mechanism for MC accumulation [73]. However, inhibition of migration was also shown for epithelial cells [74] and neutrophils [75]. Contradictory results can be based on different experimental setups or chymase concentrations.

It is known that MMPs and their inhibitors TIMPs play important roles in tissue remodeling by regulating ECM degradation, giving them a critical role in trophoblast invasion in early human pregnancy. Specifically, MMP endopeptidases degrade ECM proteins while TIMPs interfere with ECM degradation by inhibiting MMPs [25]. In the context of early pregnancy, particularly the invasion of trophoblasts during implantation and placentation, MMP2 and MMP9, as well as TIMP1, 2, and 3 are of particular importance [76,77,78]. Trophoblast invasion is mediated in an autocrine manner by trophoblast cells and in a paracrine manner by uterine factors. Cytokines, chemokines, growth factors, and hormones have been shown to regulate MMPs’ and TIMPs’ synthesis, activation, and secretion [79,80]. In our study we investigated whether HMC-1 or rhuCMA1 influence MMPs’ and TIMPs’ expression in EVTs. Our results show that rhuCMA1 did not affect TIMP3 mRNA levels in EVTs while significantly decreasing the level of TIMP3-positive cells. Additionally, the coculture of MCs and EVTs led to significantly lower TIMP2 and -3 mRNA expression levels and a significantly lower number of TIMP1- and -3-positive cells. MMPs and their inhibitors constitute to a multigene family of secreted and cell-surface enzymes. Therefore, MMPs and TIMPs were also analyzed in the supernatant of HTR-8/SVneo cells after 24 h. Due to the unexpected results with regard to TIMP1 expression, supernatants were also analyzed at 48 h and 72 h. The reason for the difference between mRNA and molecular expression could be that the samples were analyzed at different time points, because the mRNA samples were not sufficient to be measured at 24 h. Briefly, results show a decrease in TIMPs in EVTs in response to HMC-1 and rhuCMA1. The finely regulated balance of MMP and TIMP expression is important to appropriate trophoblast invasion and placentation. MMP activity can be altered by modified TIMP expression or changes in the MMP/TIMP ratio. In this context, a downregulation of TIMP expression can increase trophoblast invasion [79]. For example, it was shown in vitro that decreased TIMP-1 expression promotes the invasion of first-trimester human trophoblasts [81].

The importance of angiogenesis in proper placental development is indisputable. There is a known association between MCs and angiogenesis, but the exact role that MCs play in this process for pregnancy is still unclear. It is thought that the mediators released by MCs are important for neovascularization. Several studies, aiming to understand the way in which specific MC granule constituents induce angiogenesis, suggest that MC proteases induce tumor angiogenesis [45,82,83,84]. To investigate the effect of rhuCMA1 on angiogenesis during pregnancy, we performed a tube-formation assay with HUVECs stimulated with rhuCMA1. Our results showed that low concentrations of rhuCMA1, in particular, induce endothelial-cell angiogenesis. Higher concentrations increased parameters such as the number of nodes, segments, meshes, and master junctions, but were not statistically significant. Studies using a hamster sponge-implant model showed that chymase inhibitors reduce MC-mediated angiogenesis by interfering with the Ang-II-generating function of MCs and suppression of VEGF [45,85]. Another study indicated that chymase may induce angiogenesis by activating MMP9 to regulate ECM remodeling [86]. This suggests that chymase-induced angiogenesis might involve chymase-Ang II-VEGF or pro-MMP9-MMP9-ECM-degradation pathways. Our study presented here suggests that the MC chymase CMA1 plays a significant role for angiogenesis in a pregnancy-relevant setting.

The present study has some limitations. We performed this study with the use of cell lines and primary cells for in vitro functional research. As such, it is not known if the results can be fully translated to the in vivo situation. Additionally, we did not use chymase inhibitors in our study. Chymostatin and soybean trypsin inhibitor (SBTI) are often used as chymase inhibitors in in vitro studies. Chymostatin is an inhibitor of several chymotrypsin-like serine proteases. SBTI is a potent inhibitor of trypsin with less inhibitory action on chymotrypsin and elastase. Due to the nonspecificity of most chymase inhibitors in vitro, we decided instead to use rhuCMA1 to analyze a possible involvement of the specific MC mediator after confirming an effect of HMC-1. An approach for future studies would be the generation of a CRISPR/Cas or siRNA-mediated-gene knockout-cell line and comparison with a nonmodified-MC line.

## 5. Conclusions

In conclusion, MCs and chymase CMA1 modulate VSMCs, EVTs, and endothelial cells’ functions, and by doing so have profound effects in cellular steps that are relevant to SA remodeling. Identifying cells and molecules associated with uteroplacental and vascular remodeling could help to design new approaches to the prediction and management of pregnancy complications such as preeclampsia or premature labor.

## Figures and Tables

**Table 1:** Flow-cytometry antibodies.

Antibody	Dilution	Company
FVD-eFluor 506	1:500	Invitrogen, Karlsruhe, Germany
Cytokeratin 7-APC	1:100	R&D Systems, Wiesbaden, Germany
MMP2-PE	1:200	R&D Systems, Wiesbaden, Germany
MMP9-FITC	1:200	R&D Systems, Wiesbaden, Germany
TIMP1-AF405	1:200	R&D Systems, Wiesbaden, Germany
TIMP3-AF750	1:200	R&D Systems, Wiesbaden, Germany

**Table 2:** RT-PCR primers.

Gene	Forward Primer	Reserve Primer	UPL Number
GUSB	*cgccctgcctatctgtattc*	*tccccacagggagtgtgtag*	57
MMP2	*cggttttctcgaatccatga*	*gagtccgtccttaccgtcaa*	34
MMP9	*tcttccctggagacctgaga*	*gagtgtaaccatagcggtacagg*	27
TIMP1	*ctgttgttgctgtggctgat*	*aacttggccctgatgacg*	3
TIMP2	*gaagagcctgaaccacaggt*	*cggggaggagatgtagcac*	43
TIMP3	*ctgtgcaacttcgtggagag*	*ggcaggtagtagcaggacttg*	14

**Table 3:** Assay detection ranges.

Analyte	Detection Range (pg/mL)	Sensitivity (pg/mL)
MMP2	158.20–1400,000	
MMP9	87.80–300,000	
TIMP1	77.00–800,000	
TIMP3	156–10,000	<2
Chymase	156–10,000	<39

## Data Availability

Data is contained within the article or Supplementary Materials.

## Supplementary Materials

The following are available online at https://www.mdpi.com/article/10.3390/cells11071158/s1, Figure S1: CMA1 expression of calcium ionophore A23187-treated HMC-1 cells. Figure S2: Flow cytometry gating strategy: HTR-8 ± HMC-1 assay. Figure S3: Flow cytometry gating strategy: HTR-8 ± rhuCMA1 assay. Table S1: MMPs and TIMPs expression in supernatant of HTR-8 cells co-cultured with HMC-1 or treated with rhuCMA1.
