# Supplementary material for: Insights into Early-Pregnancy Mechanisms: Mast Cells and Chymase CMA1 Shape the Phenotype and Modulate the Functionality of Human Trophoblast Cells, Vascular Smooth-Muscle Cells and Endothelial Cells
Source: Cells. 2022 Mar 29;11(7):1158. doi: 10.3390/cells11071158 (PMC8997408; doi:10.3390/cells11071158)
Supplement: Supplementary file 1 [file cells-11-01158-s001.zip › cells-1544583-supplementary.pdf]

cells MDPI

SUPPLEMENTARY INFORMATION

Insights into early pregnancy mechanisms: mast cells and chymases CMA1 shape the phenotype and modulate the functionality of human trophoblast cells, vascular smooth muscle cells and endothelial cells.

Authors: Ningjuan Zhang[1,2], Anne Schumacher[1,2], Beate Fink[1], Mario Bauer[1], Ana Claudia Zenclussen[1,2] and Nicole Meyer[1,2]

Ningjuan Zhang [1,2], Anne Schumacher [1,2], Beate Fink [1], Mario Bauer [1], Ana Claudia Zenclussen [1,2] and Nicole Meyer [1,2,*]

1 Department of Environmental Immunology, UFZ-Helmholtz Centre for Environmental Research Leipzig-Halle, 04318 Leipzig, Germany; ningjuan.zhang@ufz.de (N.Z.); anne.schumacher@ufz.de (A.S.); beate.fink@ufz.de (B.F.); mario.bauer@ufz.de (M.B.); ana.zenclussen@ufz.de (A.C.Z.)
2 Perinatal Immunology, Saxonian Incubator for Clinical Translation (SIKT), Medical Faculty, University Leipzig, 04103 Leipzig, Germany
* Correspondence: nicole.meyer@ufz.de; Tel.: +49-341-235-1542

Fig. S1

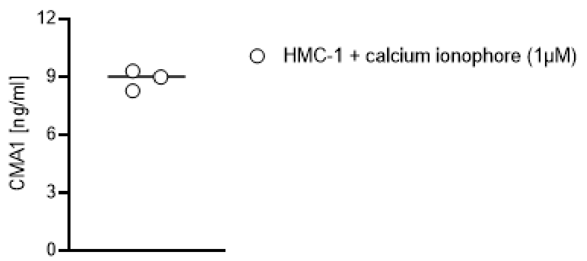

Figure S1: CMA1 expression of calcium ionophore A23187-treated HMC-1 cells. CMA1 expression (ng/ml) measured via ELISA in the supernatants of calcium ionophore A123187-treated HMC-1 cells.

Fig. S2

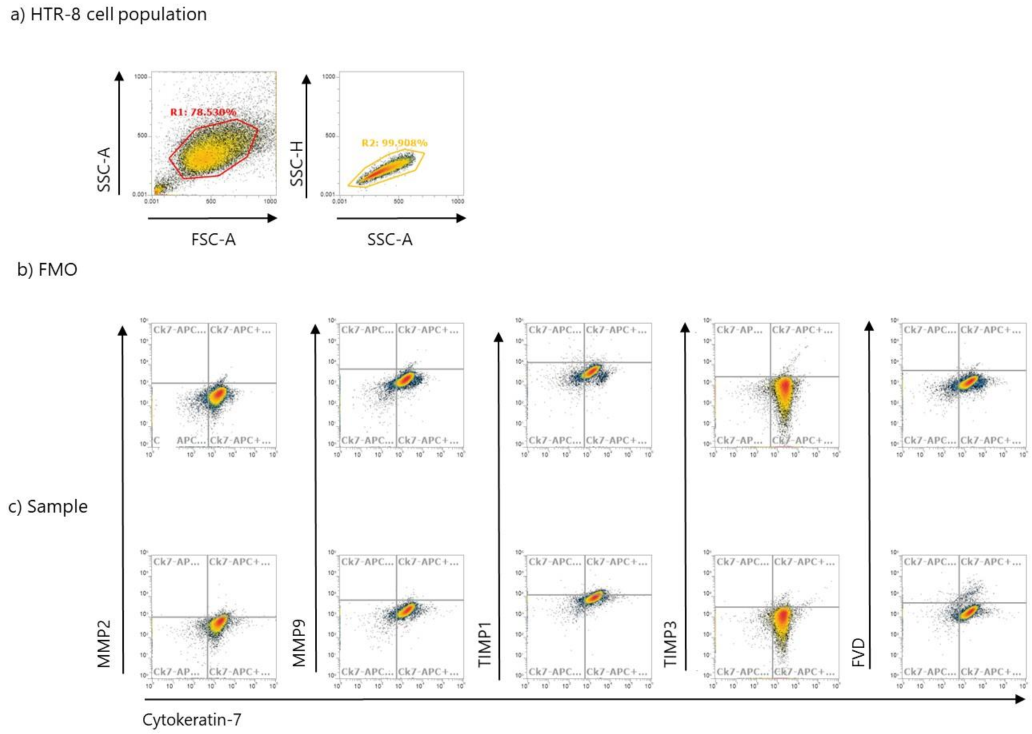

Figure S2: Flow cytometry gating strategy: HTR-8 ± HMC-1 assay. a) Representative gating strategy for HTR-8 cell population. **b)** Representative Fluorescence minus one (FMO) control or **c)** sample plots for MMP2, MMP9, TIMP1, TIMP3 or FVD of HTR-8 cells. HTR-8: HTR-8/SVneo cells; FMO; Fluorescence Minus One.

Fig. S3

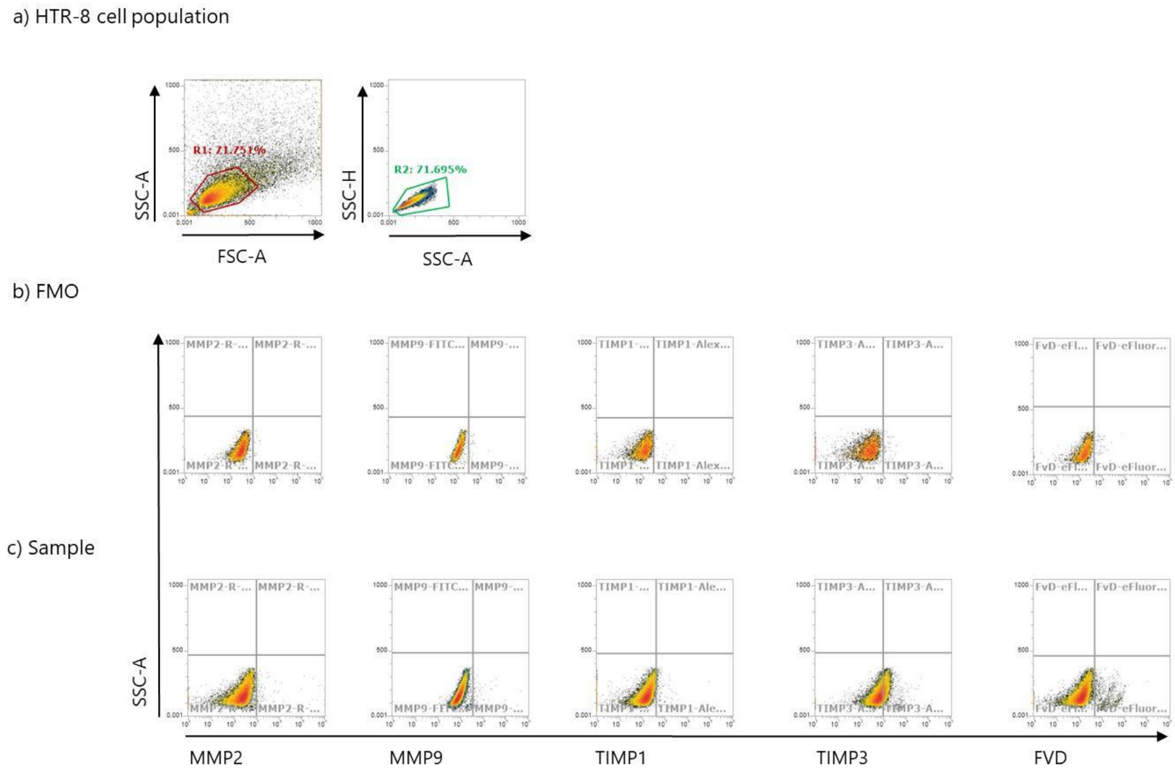

Figure S3: Flow cytometry gating strategy: HTR-8 ± rhuCMA1 assay. a) Representative gating strategy for HTR-8 cell population. **b)** Representative FMO control or **c)** sample plots for MMP2, MMP9, TIMP1, TIMP3 or FVD of HTR-8 cells stimulated ± rhuCMA1. HTR-8: HTR-8/SVneo cells; rhuCMA1: recombinant human chymase; FMO; Fluorescence Minus One.

Tab. S1

Table S1: MMPs and TIMPs expression in supernatant of HTR-8 cells co-cultured with HMC-1 or treated with rhuCMA1

Median (95%CI) (pg/ml)	TIMP1	TIMP2	MMP2	MMP9
HTR-8+HMC-1 co-culture experiment 24h				
HTR-8	7313 (5503,7767)	n.d.	n.d.	n.d.
HTR-8+HMC-1 (1:1)	13674 (9005,18731)			
HTR-8+HMC-1 (1:5)	20403 (17937,20924) **			
HTR-8+rhuCMA1 experiment 24h				
HTR-8	11049 (8497,13737)	13238 (7805,18671)	n.d.	n.d.
HTR-8+rhuCMA1 (3ng/ml)	10864 (7688,13574)	3216		
HTR-8+rhuCMA1 (30ng/ml)	10902 (6953,13330)	12446 (6222,18671)		
HTR-8+rhuCMA1 (300ng/ml)	11666 (9911,12213)	5453 (1740,7805)		
HTR-8+HMC-1 co-culture experiment 48h				
HTR-8	16751 (15959,17792)	n.d.	n.d.	n.d.
HTR-8+HMC-1 (1:1)	23235 (22960,23617)			
HTR-8+HMC-1 (1:5)	22686 (20487,25317)			
HTR-8+rhuCMA1 experiment 48h				
HTR-8	15156 (11125,25767)	12934 (1740,14777)	n.d.	n.d.
HTR-8+rhuCMA1 (3ng/ml)	15081 (9761,21313)	11158 (8619,12038)		
HTR-8+rhuCMA1 (30ng/ml)	12540 (11279,24969)	7805 (7006,8619)		
HTR-8+rhuCMA1 (300ng/ml)	13825 (10515,29370)	n.d.		
HTR-8+HMC-1 co-culture experiment 72h				
HTR-8	34065 (24455,50822)	n.d.	n.d.	n.d.
HTR-8+HMC-1 (1:1)	38357 (29390,55236)			
HTR-8+HMC-1 (1:5)	27160 (24969,44776)			
HTR-8+rhuCMA1 experiment 72h				
HTR-8	24352 (21280,30972)	32835 (16829,53159)	n.d.	n.d.
HTR-8+rhuCMA1 (3ng/ml)	24591 (23323,27159)	35269 (22821,51336)		
HTR-8+rhuCMA1 (30ng/ml)	21080 (16553,33073)	45556 (29635,50729)		
HTR-8+rhuCMA1 (300ng/ml)	25485 (22471,27420)	47862 (29817,60014) *		
